# The relationship between macro-socioeconomics determinants and COVID-19 vaccine distribution

**DOI:** 10.3934/publichealth.2021052

**Published:** 2021-09-18

**Authors:** Ali Roghani

**Affiliations:** Division of Epidemiology, University of Utah School of Medicine, USA

**Keywords:** COVID-19, immunization programs, vaccine distribution, gross domestic product, human development index, SARS-CoV-2 infection, Pfizer vaccine

## Abstract

Since coronavirus disease 2019 (COVID-19) has continued to spread globally, many countries have started vaccinations at the end of December 2020. This research examines the relationship between COVID-19 vaccine distribution and two macro-socioeconomics measures, including human development index and gross domestic product, among 25 countries for two points in time, including February and August 2021. The COVID-19 dataset is a collection of the COVID-19 data maintained by Our World in Data. It is a daily updated dataset and includes confirmed cases, vaccinations, deaths, and testing data. Ordinary Least Squares was applied to examine how macro-socioeconomic measures predict the distribution of the COVID-19 vaccine over time.

**Results:**

The results indicate that a higher gross domestic product per capita is positively associated with higher COVID-19 vaccine distribution, and this relationship becomes more robust over time. However, some countries may have more successful vaccine distribution results regardless of their gross domestic product. In addition, the result shows human development index does not have a significant relationship with vaccine distribution.

**Conclusion:**

Economic measures may be counted as a more vital indicator for vaccine distribution as they have a more direct relationship distribution with health infrastructure than social measures such as human development index.

## 1. Introduction

COVID-19 vaccine developers had an extensive racing against time to develop, test, approve and produce an effective vaccine for COVID-19. It is expected companies produce up to 9 billion doses by the end of 2021 [1]. In addition to socioeconomic factors [2]–[4], human mobility, climate conditions, lower air pollution are significant environmental and risk factors that reduce COVID-19 transmission [5]–[13]. However, COVID-19 vaccines will be the most crucial determinant in the future for decreasing morbidity and mortality and achieving herd immunity of COVID-19 [14]. To diminish the spread of the virus, there is a need to vaccinate a large world population from low to high-income countries.

The leading vaccine developers have distributed most of their products to rich countries. Lower-income countries may have to be waitlisted until 2023 due to poor administration and resources [15]–[17], which has led to an increasing global divide in vaccine distribution, and eventually, lower people's well-being. From the beginning of the pandemic, the World Health Organization demanded vaccine stockpiles be distributed equitably and created the COVID-19 Vaccines Global Access (COVAX) initiative to help accomplish this purpose [18].

There is a bidirectional relationship between countries' macro-socioeconomic status and the effects of COVID-19 [19]. Previous research indicates that sociodemographic risk factors were significantly associated with COVID-19 incidence and mortality [20] and negatively impact human life and a higher disease burden in low-income countries [17]. Lower socioeconomic status is also associated with a lower COVID-19 vaccination percentage [21]. On the other hand, the previous research indicates that the COVID-19 pandemic increases pre-existing social disparities and generates new inequality by magnifying the social distribution of economic vulnerability [22]. However, government policies, health systems, and countries' macro-socioeconomic determinants are fundamental factors to speed up the rollout of vaccines at a country level. Freed (2021) discussed two components that stand out as essential circumstances impacting the COVID-19 vaccine and immunization programs' effectiveness in both the short and long term. The two components are a national strategy for vaccine distribution and public health infrastructure [23].

Regarding national strategy for vaccine rollout, governments maintain authority and responsibilities for the ordering, paying, and distributing COVID vaccines from manufacturers, which requires rigorous testing to ensure safety. As the initial vaccine supply does not meet demand, vaccines must be distributed to individuals at the highest risk [24]. Research of national vaccine distribution strategies is challenging because only a few countries have publicly available data. In the absence of such information, Gross Domestic Product (GDP) is an indicator of countries' ability to produce and distribute vaccines. Countries with high GDP growth have a significant capital investment that provides outstanding physical and human capital investments to improve vaccination [25].

In addition to the national strategy for vaccination, public health infrastructures are essential to ensure immunization. Public health infrastructure should be well-prepared and well-staffed to take on the responsibility to deliver enough doses of vaccines in fast order [23]. These infrastructures should include plans for a COVID-19 mass vaccination program to address possible restrictions to vaccine acceptance by applying cultural determinants. Additionally, health care providers should develop COVID-19 vaccine educational campaigns through social media and present solid recommendations for COVID-19 vaccination [24]. These campaigns and educations are essential contributors to reduce vaccine hesitancy. In many countries, people are concerned about vaccine safety as the vaccines had been bought or developed quickly [26]. Additionally, there is a continuing misinformation campaign against COVID-19 vaccines on social media [27], which is higher for developing countries, particularly among religious and ethnic groups [28]. To assess public health infrastructures, the Human Development Index (HDI) is applied in this research, which is an index of life expectancy, education, and per capita income to evaluate countries' social health determinants and health care resources [29]. Higher HDI can be associated with high investments in the health sector, leading to more positive social conditions to apply shorter lockdowns with lower adverse effects on countries' socioeconomic status [3]. As trust in governments and public health authorities influence vaccine acceptance [30],[31], higher HDI and more enhanced people's well-being may reduce the growing problem of vaccine hesitancy and assist countries in vaccinating people in a shorter time.

This research examines the relationship between GDPs per capita, HDI, and the distribution of COVID-19 vaccine among 25 countries in February 2021 and August 2021 to understand how macroeconomics measures impact people's vaccination rates among low to high-income countries. These two measures were proposed to estimate countries' capability to maximize vaccination distribution to prevent new pandemic threats. The idea here is to incorporate the multivariate indicators of macroeconomics measures to understand the general ability of different countries to cope with COVID-19 threats in different pandemic stages. These variations in countries at two-point of time allow me to test the consistency of GDP, HDI, and higher vaccination rates over time. This study was designed to 1) assess the association between higher GDP, HDI, and vaccine distribution, and 2) how these associations changed over time? It is expected countries with higher GDP and HDI are more likely to have higher vaccination distribution, and these associations will be more robust in August 2021 than February 2021.

## 2. Materials and methods

### 2.1. Sample and data

The data set was built from 25 countries, accounting for approximately 33% of the world population. Data of COVID-19 vaccination were obtained from the Our World in Data website [32], which provides a public aggregated global dataset on distributed vaccinations. It includes the entire period from 13 December 2020 which was the first vaccination data that has been updated daily ever since. This dataset consists of the cumulative number of COVID-19 vaccinations in these 25 countries and calculated daily vaccination rates and population-adjusted statistics. Data on socioeconomic measures were obtained from the World Bank [33],[34]. The combination of these two datasets is essential to examine how socioeconomic measures at a national level predict vaccine rollouts relative to the population in these 25 countries.

### 2.2. Measures of variables

GDP per capita, calculated in US dollars, was used as an indicator of standard of living. HDI is a composite index measure for the general evaluation of human development status that shows the extent of addressing the three primary aspects of development: life expectation, education, and living standard measures. The outcome is the number of people vaccinated per hundred in February 2021 and August 2021. There is one year lag between independent variables (i.e., GDP per capita and HDI), and the outcome was mainly estimated in 2021. Twenty-five countries were included in this study that is mostly from high-income countries. Health indicators (i.e., handwashing facilities and hospital beds) are government responses to the pandemic used to control the analyses. In addition, life expectancy, population density and extreme poverty were used to adjust the models to assess countries' sociodemographic determinants.

### 2.3. Model and data analysis procedure

Ordinary Least Squares (OLS) was used to measure the associations between macro-socioeconomic variables (i.e., GDP and HDI) and outcome variables (i.e., people vaccinated per hundred in February and August). The odds ratios for each explanatory variable with the *P-value* were presented at the 95% confidence interval to estimate the precision of the odds ratios. The associations were estimated for two points in time, including two models (i.e., unadjusted and adjusted). Models 1 and 2 were included information for February 2021, and Model 3 and 4 were for August 2021. The data were analyzed with the R programming language (version 3.5.2) (R Core Team 2018).

## 3. Results

**Table 1.** Descriptive result.

Country	Human Development Index	GDP per Capita	People Vaccinated per Hundred (February)	People Vaccinated per Hundred (August)
Austria	0.908	45436.69	2.68	58.86
Belgium	0.916	42658.58	2.76	69.48
Bulgaria	0.813	18563.31	0.42	14.81
Canada	0.926	44017.59	2.17	71.54
Chile	0.843	22767.04	0.30	72.35
Czechia	0.888	32605.91	2.28	52.79
Denmark	0.929	46682.51	3.11	72.73
Estonia	0.871	29481.25	2.17	48.32
France	0.901	38605.67	2.52	62.03
Germany	0.936	45229.25	2.36	61.34
Indonesia	0.694	11188.74	0.20	17.36
Israel	0.903	33132.32	36.80	66.87
Italy	0.880	35220.08	2.30	63.76
Latvia	0.847	25063.85	0.92	40.87
Lithuanian	0.858	29524.26	2.70	52.00
Malta	0.878	36513.23	5.55	90.53
Mexico	0.774	17336.47	0.49	36.99
Norway	0.953	64800.06	2.05	66.34
Poland	0.865	27216.44	2.64	48.50
Romania	0.811	23313.20	3.11	25.98
Slovakia	0.855	30155.15	2.71	41.69
Slovenia	0.896	31400.84	2.65	44.30
Switzerland	0.944	54225.45	7.78	54.00
United Kingdom	0.922	39753.24	14.21	68.99
United States	0.924	54225.45	7.78	57.26

Table 1 indicates the summary of measures that are used in this study. Indonesia had the lowest GDP per Capita in the sample. Most of the European countries had a range of $30,000 to $45,000 GDP per Capita. Norway and Switzerland were on top among the sample with $64,800 and $54,225 GDP, respectively. HDI's ranged between 0.69 (Indonesia) and 0.936 (Germany). Most European countries with an average to high GDP had great progress in six months of vaccinations. In February, most of them had a lower than 10 percent vaccinated people, while in August, they vaccinated more than 60 percent of their residents. However, Bulgaria and Indonesia did not have a good performance, and they vaccinated less than 20 percent of their population.

**Figure 1.**
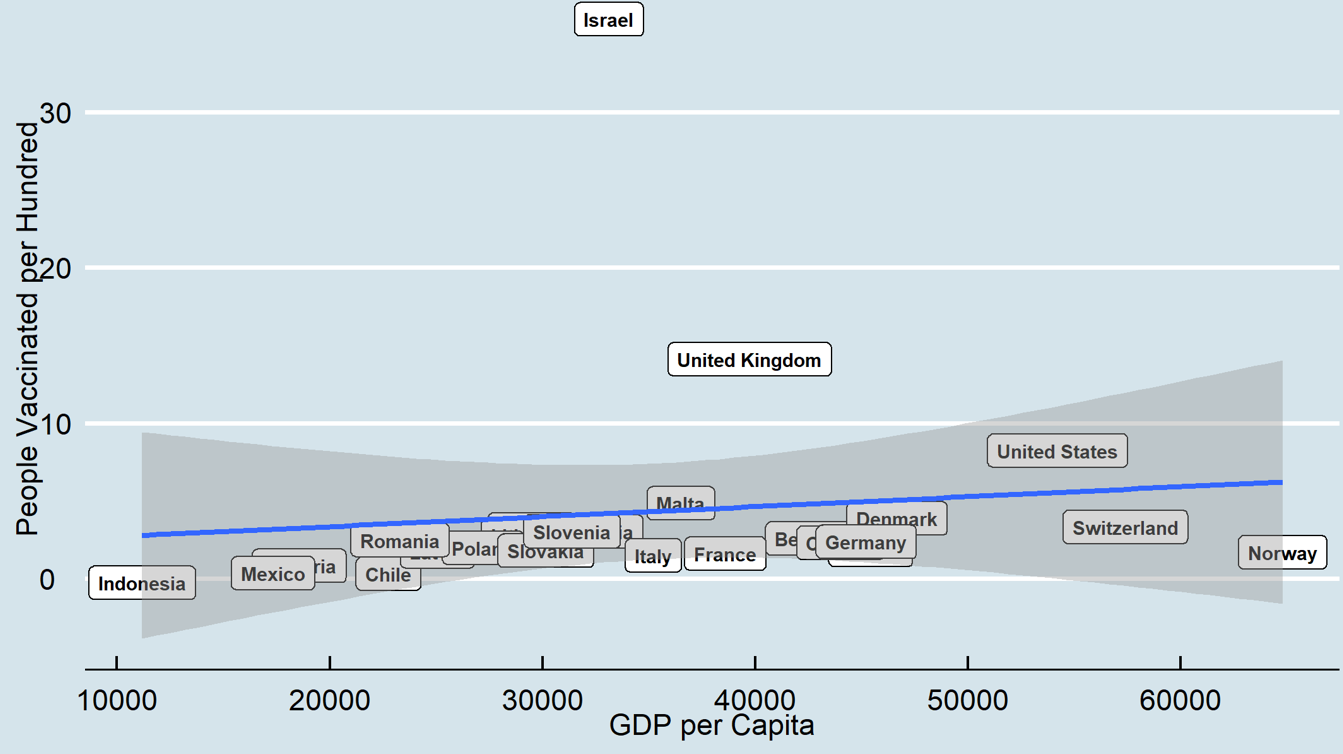
Vaccination by GDP per Capita (February 2021).

Figure 1 shows that the relationship between GDPs per capita and people vaccinated per hundred (February) is linear; however, some countries have different performances. Indonesia, with low GDP, has the most inadequate vaccination distribution. Most European countries and the United States followed the linear relationship of GDP and vaccination distribution in the first round. However, with very high GDP per capita, Norway and Switzerland had a similar performance to other European countries such as Germany and Denmark. On the other hand, the United Kingdom has a considerably high vaccination distribution than other countries with similar GDP per Capita.

**Figure 2.**
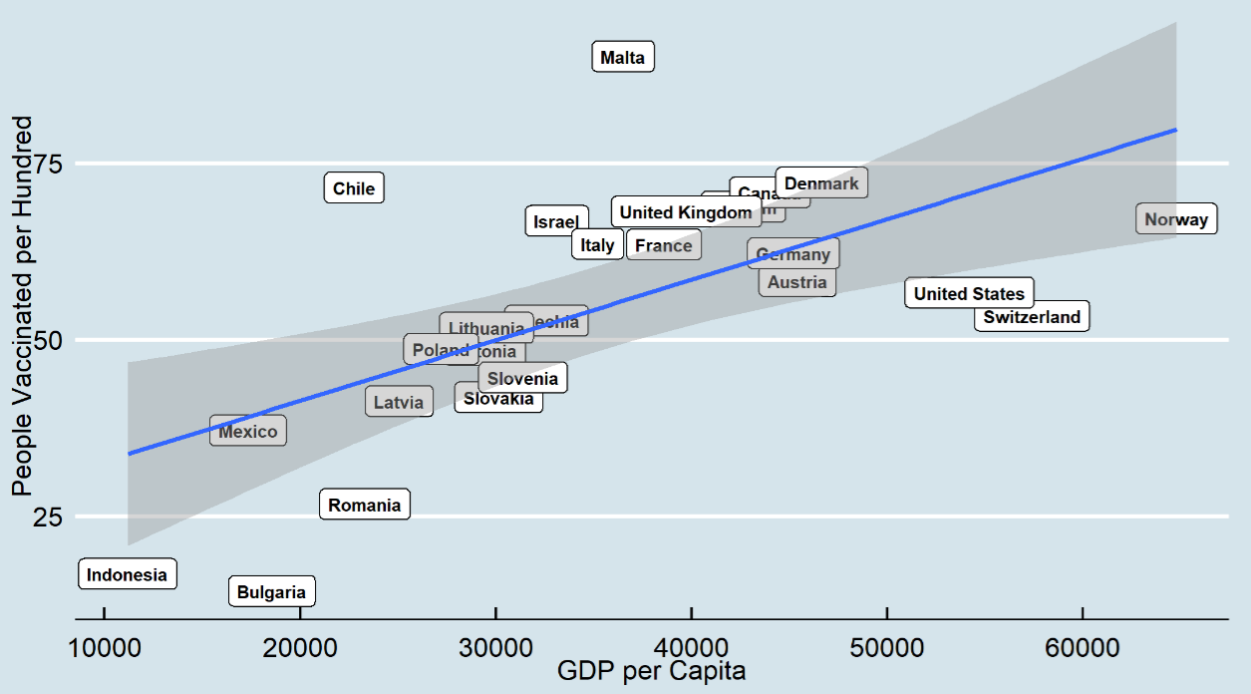
Vaccination by GDP per Capita (August 2021).

Figure 2 shows the association between GDP and vaccination in August, which suggests more variation in countries' vaccinations. Malta had an outstanding performance, and more than 90 percent of its population has been vaccinated. Romania, Bulgaria, and Indonesia had the lowest vaccination rate. Most countries followed the linear pattern, and the slope was considerably higher in the second round than in the first round. The United States showed lower performance in the second round compared to the first round. Norway and Switzerland still had lower vaccinated rates based on their GDP.

Table 2 shows the effects of GDP on vaccination rates becomes higher, 41% and 54%, respectively, in February than August (Model 1 and Model 3). The second period showed a stronger association between GDP and vaccination, and even with adjusting the models, the relationship remained consistent. The analysis shows no significant relationship between HDI and vaccination in the first period, while in the second period (August 2021), higher HDI was associated with 31% higher vaccination (Model 3). However, adjusting the model in the second period indicated no significant relationship between HDI and vaccination rate. Extreme poverty and life expectancy as a predictor of sociodemographic status had a significant association with vaccine distribution. Lower extreme poverty was associated with 28% and 26% higher vaccination rates in February 2021 and August 2021. Also, higher life expectancy was associated with 32% and 36% higher vaccination in Model 3 and Model 4, respectively. There is no association between handwashing facilities; however, countries with higher hospital beds are more likely to vaccinate their residents at 13% and 17% in February 2021 and August 2021, respectively.

**Table 2.** Logistic regression models.

	February 2021		August 2021	
	Model 1	Model 2	Model 3	Model 4
GDP per Capita	1.41**	1.37*	1.54**	1.48*
Human Development Index	1.21	1.16	1.34*	1.31
Hospital bed		1.13*		1.17*
Population density		1.13		1.13
Extreme poverty		1.28*		1.26*
Handwashing facilities		1.10		1.08
Life expectancy		1.32*		1.36*
Pseudo-R square	0.32	0.28	0.34	0.27

Notes: OR: Odds Ratios; Significant at *p  <  0.05. **p  <  0.01. ***p  <  0.001.

## 4. Discussion

This research suggests that higher GDP per capita is significantly associated with greater vaccinations, and this association becomes more robust over time. Although there was no significant relationship between HDI and vaccine distribution in the first point, higher HDI was associated with higher vaccination rates in August. However, by controlling for other factors, HDI did not have a significant relationship with vaccine distribution. The findings highlight that GDP may be more crucial than the HDI in the initial stages of critical moments like the COVID-19 pandemic for vaccine distribution. The reasoning would be that GDP may support higher production, test, and distribution. A more robust association in August indicates that the influence is considerably greater after six months, highlighting the importance of GDP in higher vaccination rates over time. Higher GDP can speed up vaccination in the first stages of vaccine rollout due to the higher association between GDP, national strategy, and resources, and assures mass vaccinations of groups at the highest risk of getting COVID-19, seriously ill individuals, and older populations. Nevertheless, HDI had a more direct relationship with public health infrastructure and social determinants, which may support a higher vaccination rate in the long term.

Although GDP per capita and vaccine distribution had a linear relationship, there is, however, a slight variation in some countries' performance. These differences can be related to their health policies, priorities, and other medical interventions. Rich countries usually have more reliable infrastructure to provide vaccination for their citizens to facilitate a mass vaccination [35]. Some countries like Norway with high GDP per capita, had a lower vaccination distribution rate, and this can reflect concerns other than medical facilities. Torjesen (2021) study shows doctors in Norway were told to evaluate severely frail old patients after receiving the Pfizer vaccine against COVID-19, following the deaths of 23 cases shortly after taking the vaccine [36]. Additionally, vaccine hesitancy is considered a vital barrier for mass vaccination [37], which has been seen in countries with different socioeconomic status [38]–[42]. The research among the French working-age shows 29.4% of them were likely to refuse COVID-19 vaccination ranging from 9.3% to 43.2% depending on vaccine characteristics [43]. It was not possible in this research to examine vaccine hesitancy. However, consistent with the current study result, no significant relationship has been found between HDI — an important indicator of education at the macro-level (i.e., mean years of schooling and expected years of schooling) — and vaccinee distribution. Moreover, lower vaccination rates in the US compared to the first round can be related to vaccine hesitancy among US citizens [24]. Previous research also shows no association between education and vaccine hesitancy. Hence, other indicators are needed to understand socio-cultural influences on vaccine distribution at the individual levels [14],[44]–[46].

In addition to GDP and HDI, health and sociodemographic indicators showed meaningful relationships with vaccination. Although previous research shows that higher population areas are more likely to have higher COVID-19 cases, the current study indicates no relationship between COVID-19 vaccines and population density [47]. Consistent with GDP's results, extreme poverty had a significant association implying the importance of financial resources for higher vaccination rates [48]. Also, higher bed hospitals show higher vaccinations indicating how essential public health resources are necessary to support lower morbidity and mortality.

Although this study shows essential implications regarding the relationship between economic, social determinants and vaccine distribution, it has limitations. This dataset was limited to a few economic, health, and social determinants. Information about laboratories, vaccine producers, and their relationship with these countries could enrich the data. Also, the availability of a vaccine is inadequate to support broad immunological protection. The vaccine requires universal acceptance by the health administrations and the public. The data used in this study did not have information on vaccine hesitancy, which is a significant obstacle to vaccination as well as the successes from people's protection [24].

## 5. Conclusions

Although countries with high GDP vaccinated a considerable percentage of their residents, wider availability of the COVID-19 vaccine in low GDP countries will play a vital role in achieving global immunity against this deadly virus. Apart from distributing more vaccines, it is also essential to ensure appropriate management of vaccines by addressing the contributing cultural and social factors. Using existing resources can help decrease the difficulties facing low GDP nations and lead the world closer to the end of COVID-19. Future studies should consider socio-cultural influences on vaccine distribution at individual levels.
